# Ischemic Postconditioning (IPostC) Protects Fibrotic and Cirrhotic Rat Livers after Warm Ischemia

**DOI:** 10.1155/2019/5683479

**Published:** 2019-06-09

**Authors:** Julia Schewe, Marie-Christine Makeschin, Ingrid Liss, Doris Mayr, Jiang Zhang, Andrej Khandoga, Simon Rothenfußer, Max Schnurr, Alexander L. Gerbes, Christian J. Steib

**Affiliations:** ^1^Berlin Institute of Health Charité – Universitätsmedizin Berlin, Germany; ^2^Department of Pathology, University of Munich, Campus Grosshadern, Munich, Germany; ^3^Department of Medicine II, University Hospital, Liver Centre Munich, LMU Munich, Germany; ^4^Department of Surgery, University of Munich, Campus Grosshadern, Munich, Germany; ^5^Division of Clinical Pharmacology, University of Munich, Campus Innenstadt, Munich, Germany

## Abstract

**Background:**

Decreased organ function following liver resection is a major clinical issue. The practical method of ischemic postconditioning (IPostC) has been studied in heart diseases, but no data exist regarding fibrotic livers.

**Aims:**

We aimed to determine whether IPostC could protect healthy, fibrotic, and cirrhotic livers from ischemia reperfusion injury (IRI).

**Methods:**

Fibrosis was induced in male SD rats using bile duct ligation (BDL, 4 weeks), and cirrhosis was induced using thioacetamide (TAA, 18 weeks). Fibrosis and cirrhosis were histologically confirmed using HE and EvG staining. For healthy, fibrotic, and cirrhotic livers, isolated liver perfusion with 90 min of warm ischemia was performed in three groups (each with n=8): control, IPostC 8x20 sec, and IPostC 4x60 sec. additionally, healthy livers were investigated during a follow-up study. Lactate dehydrogenase (LDH) and thromboxane B_2_ (TXB_2_) in the perfusate, as well as bile flow (healthy/TAA) and portal perfusion pressure, were measured.

**Results:**

LDH and TXB_2_ were reduced, and bile flow was increased by IPostC, mainly in total and in the late phase of reperfusion. The follow-up study showed that the perfusate derived from a postconditioned group had much less damaging potential than perfusate derived from the nonpostconditioned group.

**Conclusion:**

IPostC following warm ischemia protects healthy, fibrotic, and cirrhotic livers against IRI. Reduced efflux of TXB_2_ is one possible mechanism for this effect of IPostC and increases sinusoidal microcirculation. These findings may help to improve organ function and recovery of patients after liver resection.

## 1. Introduction

Different pathological processes in liver tissue such as abscesses, cysts, or benign and malignant tumours necessitate partial resection of the liver. Because of the interruption in blood flow during the surgical procedure, the residual liver suffers from ischemia reperfusion injury (IRI), which is caused by microcirculation disturbances, inflammatory processes, and reactive oxygen species [1].

To date, different methods have been investigated to reduce IRI after warm ischemia. One possibility method is pharmacological intervention, e.g., antioxidants (tocopherol) and steroids (prednisolone) used to treat ROS and inflammatory processes [2–4]. However, the administration of some substances is limited by the resulting side effects. A surgical method to protect the liver against IRI ischemic preconditioning (IPC) has been investigated. The principle of this method is to switch between short periods of ischemia and reperfusion before the main ischemia period [5]. Although clinical studies have shown decreased enzyme markers, no considerable benefit for patients after liver resection has been detected [6].

Another surgical method derived from IPC is ischemic postconditioning (IPostC), which was first described in 2002 [7]. This strategy is defined as a series of brief periods of ischemia and reperfusion applied immediately after the ischemic period before continuous reperfusion. The first experimental studies of IPostC were performed in isolated perfused hearts and showed protective effects on the myocardium [8]. Human clinical studies describe opposing results: some studies showed that IPostC may be effective in clinical practice [9, 10]; other studies could not determine any significant benefit for the patients [11, 12]. Among other organs, such as brain, kidney, and lung, IPostC was applied to the liver. The first study in 2004 described the reduction of hepatocellular apoptosis through the downregulation of Bcl-2 and the inhibition of ROS by IPostC [13]. Further investigation regarding IPostC after ischemia has shown a protective effect on liver regeneration [14] and gene expression profiles in liver tissues [15]. To date, no human studies showing clinical benefit have been performed.

Because the IPostC intervention takes place after ischemia, it presumably uses the early phase of reperfusion as its therapeutic target [16]. A very important and limiting aspect during ischemia and at the beginning of reperfusion is the limited blood flow in liver sinusoids. In addition to shifted conditions of nitric oxide (NO) and endothelin, thromboxane A_2_ (TXA_2_) leads to a narrowing of the liver sinusoids with impaired microcirculation [17, 18]. Furthermore, vasoconstrictor TXA_2_ contributes to portal hypertension in fibrotic and cirrhotic livers [19]. Thus, we were interested in whether IPostC can influence microcirculation in the liver by regulating TXA_2_.

The protective effect of IPostC has been demonstrated in healthy livers but data exist neither for fibrotic livers nor for cirrhotic livers. However predamaged livers are thought to be more vulnerable to IRI than healthy livers. Therefore, we investigated the influence of IPostC on two different liver models. From a clinical perspective, this intervention may help to reduce complications and to improve graft function after liver resection.

## 2. Materials and Methods

### 2.1. Animal Studies

During this study, animals were ethically treated according to the criteria established by the National Academy of Sciences and published by the National Institutes of Health, as well as to the legal requirements of Germany. All animal experiments were approved by the local government (Regierung von Oberbayern, Munich, Germany) and were reported to the responsible authorities.

### 2.2. Bile Duct Ligation

To induce liver fibrosis, male Sprague-Dawley rats (151-175 g) were anaesthetized using an intraperitoneal injection of pentobarbital (diluted 1:3 with NaCl, 30-50 mg/kg of body weight). Additional analgesia included a subcutaneous injection of buprenorphine (0.03-0.05 mg/kg of body weight) that was administered 24 h before anaesthesia, during anaesthesia and during the five-day postoperative period. After a midline laparotomy, the liver was upward to properly visualize the portal system. The bile duct was traced with a pair of tweezers, ligated twice with 3-0 PROLENE (ETHICON LLC, Somerville), and cut between the ligations. The liver was pulled back, and the abdomen was closed with two continuous sutures (3-0 PROLENE). To induce a fibrotic liver, the perfusion followed after 4 weeks. Until the perfusion the rats received vitamin K (0.4 mg/kg of body weight) three times per week in order to prevent bleeding.

### 2.3. TAA Treatment

Liver cirrhosis was induced in male Sprague-Dawley rats by administration of thioacetamide (TAA) via drinking water during a period of 18 weeks. The animals were weighed once per week, and the doses of TAA that they received were adjusted according to body weight [20].

### 2.4. Isolated Rat Liver Perfusion

**Figure 1:**
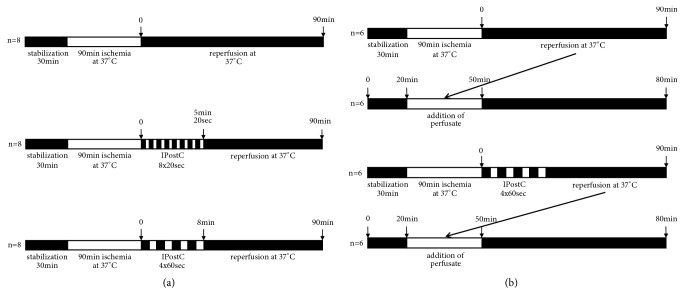
*Experimental protocols for animal studies*. (a) Male SD rats underwent bile duct ligation over 4 weeks to induce liver fibrosis or received thioacetamide via drinking water over 18 weeks to induce liver cirrhosis. For the implementation of warm ischemia, healthy, fibrotic, and cirrhotic livers were perfused for 30 min, stored for 90 min at 37°C, and reperfused for 90 min. The IPostC intervention groups included 8x20 sec and 4x60 sec cycles that were performed after ischemia (control and intervention groups, each n=8). (b) For the follow-up study, we performed warm ischemia in healthy livers of male SD rats (postconditioned with IPostC 4x60 sec and nonpostconditioned, each n=6) and collected the reperfusion perfusate to perfuse a second healthy liver for 80 min.

The male SD rats were anaesthetized using an intraperitoneal injection of pentobarbital (30-50 mg/kg of body weight). The rats intravenously received heparin (2000-5000 U/kg of body weight) via the femoral vein. After midline laparotomy, one ligature was placed around the abdominal inferior caval vein above the renal artery, and two ligatures were placed around the portal vein at a distance of approximately 1 cm. Then, a soft catheter was inserted into the portal vein and secured with the previous ligatures. To ensure effluent perfusate, the abdominal inferior caval vein was cut below the renal artery. After this step, the thorax was opened, and two ligatures were placed around the thoracic inferior caval vein above the level of the liver. A soft catheter was inserted into the right atrium and secured using ligatures. The ligature around the abdominal inferior caval vein was drawn and media could be collected through the atrium. Finally, the common bile duct was cannulated with a catheter to determine the level of bile flow during the perfusion. The portal perfusion pressure was continuously monitored. In all experiments, the liver was perfused with Krebs-Henseleit buffer (KH-buffer, 37°C, pH 7.4), which had been gassed with carbogen (95% O_2_, 5% CO_2_) using an oxygenator. To induce warm ischemia, the flow of KH-buffer was interrupted for 90 min at 37°C. For the control group (n=8), only 90 min reperfusion was performed. For the intervention groups (each with n=8) two different cycles of IPostC were tested immediately after ischemia: 8x20 sec and 4x60 sec (Figure 1(a)).

### 2.5. Follow-Up Study

In this variation of the isolated rat liver perfusion, we induced warm liver ischemia in healthy livers of two groups of rats (postconditioned with or without IPostC 4x60 sec, each with n=6) and collected their reperfusion perfusate, which we subsequently used to perfuse a second healthy rat liver (Figure 1(b)).

### 2.6. Lactate Dehydrogenase Measurement

An enzymatic test of the kinetic indicator reacting was used to measure the quantity of LDH in the perfusate. LDH activity is proportional to the reduction of NAD to NADH. Under NAD catalysis, lactic acid is converted to pyruvate by LDH. The enzymatic activity of LDH is proportional to the rate of NADH production. The amount of NADH produced is quantified by increase in absorbance at 365 nm. The concentration of LDH (in mU/min x g liver) was calculated according to the linear range after measurement [21].

### 2.7. Enzyme-Linked Immunosorbent Assay

The efflux of thromboxane B_2_ (TXB_2_) into the perfusate was measured using an enzyme-linked immunosorbent assay (Cayman Chemical, Ann Arbor, Michigan, USA).

### 2.8. Histological Evaluation

Sections of the livers were fixed in 4% buffered formalin, dehydrated in graded ethanol, and embedded in paraffin. These sections were stained with HE (haematoxylin/eosin) and EvG (Elastica-van-Gieson) and histologically evaluated (Table 1) using the following parameters: fibrosis, inflammation, bile duct proliferation, fat deposition, and group necroses. The parameters were graded as follows: 0=not at all, 1=low, 2=moderate, and 3=severe.

### 2.9. Drugs and Reagents

TAA was obtained from Sigma Aldrich Co. (St. Louis, USA).

### 2.10. Statistical Analysis

All data are expressed as the mean ± standard error of the mean (SEM). Normality of data distribution was tested by Pearson's chi-squared test and Shapiro-Wilk test. Statistical analysis of the data was performed using two-sample Student's t-test for normally distributed data and independent samples with continuous measurements. A value of p<0.05 was considered statistically significant.

## 3. Results

### 3.1. Histological Evaluation after Warm Ischemia

**Figure 2:**
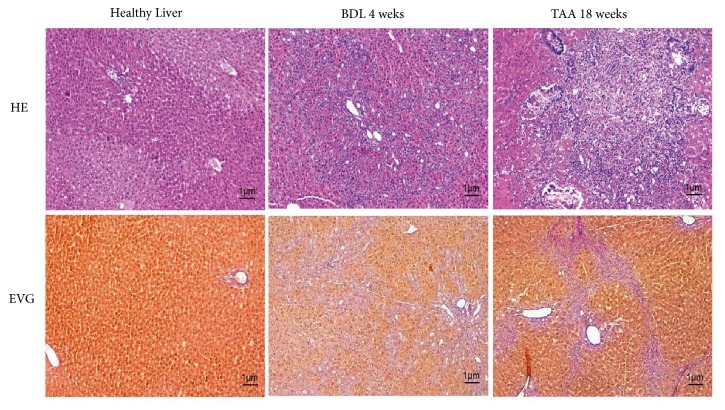
*Histological evaluation of healthy, fibrotic, and cirrhotic livers after warm ischemia*. After the induction of liver fibrosis in male SD rats using bile duct ligation (BDL) and the induction of liver cirrhosis using thioacetamide (TAA) administered via drinking water, healthy (a/b), fibrotic (c/d), and cirrhotic (e/f) livers were perfused (100x magnification). A stabilization period of 30 min was followed by 90 min of storage at 37°C and 90 min of reperfusion. For the IPostC intervention groups, 8x20 sec and 4x60 sec cycles were performed after ischemia (control and intervention groups, each with n=8). After the experiments, liver samples were fixed in 4% formalin and histologically evaluated (Table 1).

The healthy livers showed low inflammation with single cell necrosis, apoptosis, and mitoses, as well as diffuse low to moderate group necrosis. The cells had no clear core loss, but colour loss and loosened cytoplasm were observed (Figures 2(a) and 2(b)).

In BDL livers, there was low to moderate fibrosis with septa in the area of bile duct proliferation and also low to moderate inflammation with single cell necrosis, apoptosis, and mitoses. The high bile duct proliferation was diffusely distributed throughout the parenchyma (Figures 2(c) and 2(d)).

After TAA and 90 min warm ischemia, we observed low to moderate fibrosis with fibroblast proliferation and focally confluent septa and low to moderate inflammation. These changes were mixed with predominantly chronic focal purulent-destructive cholangitis with single cell necrosis, apoptosis, and mitoses. Furthermore, we observed low to moderate bile duct proliferation accompanied by the accumulation of vascular and fibroblast proliferation, as well as low focal group necrosis with connective tissue remodelling (Figures 2(e) and 2(f)).

### 3.2. Cell Damage, Organ Function, and Portal Pressure

**Figure 3:**
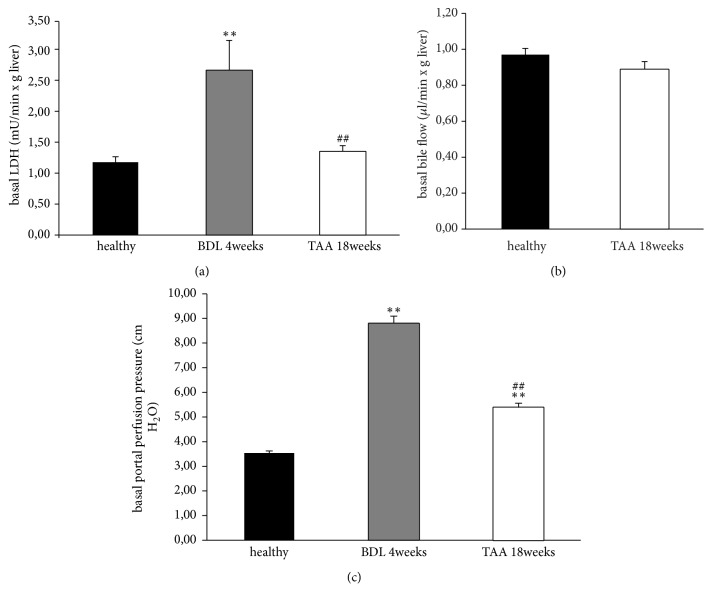
*Basal values of lactate dehydrogenase, bile flow, and portal perfusion pressure in healthy, fibrotic, and cirrhotic livers*. To induce liver fibrosis, bile duct ligation (BDL) was performed in male SD rats, and to induce liver cirrhosis, animals received thioacetamide (TAA) administered via drinking water. Healthy, fibrotic, and cirrhotic livers were perfused for 30 min. Lactate dehydrogenase (LDH; (a)) was measured in the perfusate, bile was collected to determine the bile flow (b), and the portal perfusion pressure (c) was monitored continuously. All data are expressed as the mean ± SEM. Significant values are specified as *∗∗*p<0.01 (fibrotic/cirrhotic liver compared to healthy liver) and ^##^p<0.01 (cirrhotic liver compared with fibrotic liver).

Basal values of lactate dehydrogenase (LDH), bile flow, and portal perfusion pressure are shown in Figures 3(a)–3(c). As shown in the figure, BDL animals showed much higher basal levels of LDH (Figure 3(a)) than animals with healthy livers and TAA animals. Furthermore, both liver disease models BDL and TAA had a higher baseline for portal perfusion pressure (Figure 3(c)) compared with healthy livers.

**Figure 4:**
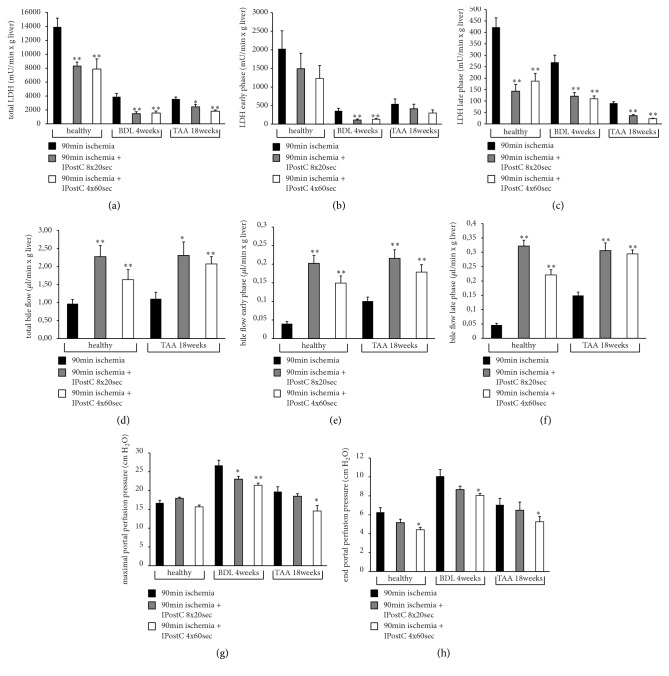
*Lactate dehydrogenase, bile flow, and portal perfusion pressure in healthy, fibrotic, and cirrhotic livers after warm ischemia ± IPostC*. Liver fibrosis was induced in male SD rats using bile duct ligation (BDL) over 4 weeks. Liver cirrhosis was induced using thioacetamide (TAA) administered via drinking water over 18 weeks. The healthy, fibrotic, and cirrhotic livers were perfused for 30 min, followed by 90 min of ischemia at 37°C and 90 min of reperfusion. The IPostC intervention groups included 8x20 sec and 4x60 sec cycles that were performed after ischemia (control and intervention groups, each n=8). Lactate dehydrogenase (LDH) was measured in the perfusate and is represented as total value (a) and during the early phase (minute 0 to 40; (b)) and the late phase (minute 50 to 90; (c)) of reperfusion. Bile was collected and also the bile flow is shown as total value (d) and at the early (e) and late (f) phase of reperfusion. For the portal perfusion pressure, which was monitored continuously, the maximum (g) and the value at the end of perfusion (h) are displayed. All data are expressed as the mean ± SEM, and significant values are specified as *∗*p<0.05 and *∗∗*p<0.01.

After warm ischemia, LDH was significantly reduced by using IPostC (8x20 sec and 4x60 sec) in total (Figure 4(a)) and during the late phase (50 to 90 minutes) of reperfusion (Figure 4(c)) of the healthy livers, the BDL livers, and the TAA livers. Bile flow was significantly increased using IPostC (8x20 sec and 4x60 sec) after ischemia and reperfusion in total (Figure 4(d)) and during both the early (0 to 40 minutes) and the late phases of reperfusion (Figures 4(e) and 4(f)). Maximal portal perfusion pressure (Figure 4(g)) could be greatly reduced in BDL livers using IPostC with both interventions and in TAA livers with the 4x60 sec intervention. At the end of reperfusion (Figure 4(h)), portal perfusion pressure was significantly decreased using IPostC (4x60 sec) in all three liver models. Normality test results confirmed the normality distribution of portal perfusion pressure and LDH values collected by different time points after ischemia (Supplementary Tables 1A and 1B).

### 3.3. Follow-Up Study

**Figure 5:**
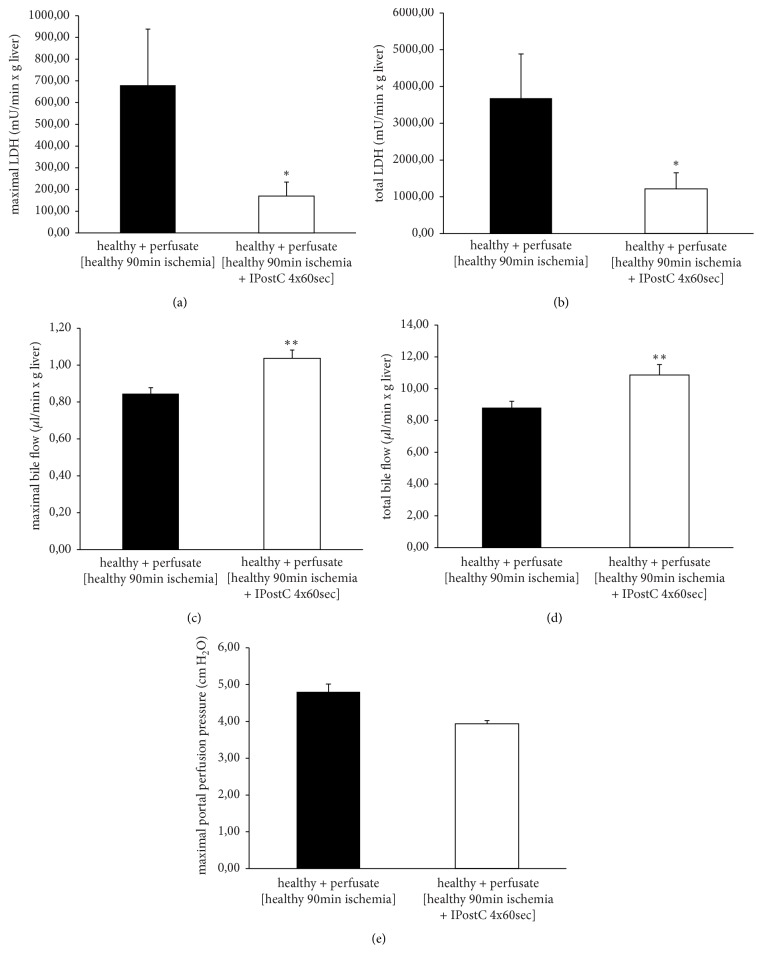
*Lactate dehydrogenase, bile flow, and portal perfusion pressure in healthy livers after follow-up study ± IPostC*. Warm ischemia in situ ± IPostC 4x60 sec was performed in the healthy livers of male SD rats. The perfusate of reperfusion was collected and subsequently used to perfuse additional healthy livers (postconditioned and nonpostconditioned, each with n=6). Lactate dehydrogenase (LDH) was measured in the perfusate and bile was collected during the time of perfusion. Both LDH and bile flow are shown as maximal (LDH (a); bile (c)) and total (LDH (b); bile (d)) values. The portal perfusion pressure (e) was monitored continuously and is represented as maximal value. Data are expressed as the mean ± SEM. Significant values are specified as *∗*p<0.05 and *∗∗*p<0.01.

Maximal and total values of LDH (Figures 5(a) and 5(b)) were much lower, and bile flow was increased (Figures 5(c) and 5(d)) after perfusion with postconditioned (4x60 sec) perfusate. Portal perfusion pressure (Figure 5(e)) was nearly equal for the postconditioned and the nonpostconditioned perfusate treated group.

### 3.4. Production of Vasoconstrictor TXB_2_

**Figure 6:**
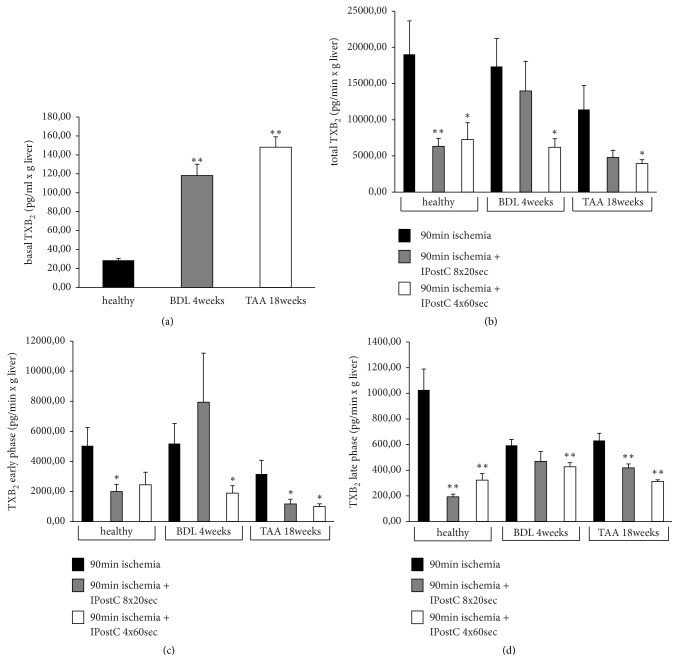
*Thromboxane B*_*2*_ *in healthy, fibrotic, and cirrhotic livers after warm ischemia ± IPostC*. The induction of liver fibrosis using bile duct ligation (BDL) over 4 weeks or the induction of liver cirrhosis using thioacetamide (TAA) administered via drinking water over 18 weeks in male SD rats was performed. The healthy, fibrotic, and cirrhotic livers were perfused for 30 min and stored at 37°C for 90 min, followed by reperfusion for 90 min. After ischemia, IPostC interventions using 8x20 sec and 4x60 sec (control and intervention groups, each with n=8) were carried out. Thromboxane B_2_ (TXB_2_) was measured in the collected perfusate using an enzyme-linked immunosorbent assay. The values for TXB_2_ are shown as basal values (a), in total (b) and during the early phase (minute 0 to 40; (c)) and the late phase (minute 50 to 90; (d)) of reperfusion. All data are expressed as the mean ± SEM. Significant values are specified as *∗*p<0.05 and *∗∗*p<0.01.

Compared to healthy livers, BDL and TAA livers showed significantly higher baseline values (Figure 6(a)) for TXB_2_. After ischemia and reperfusion, total TXB_2_ (Figure 6(b)) was significantly decreased using IPostC in healthy livers following both interventions, and in BDL and TAA livers only with the 4x60 sec intervention. The early phase of reperfusion (Figure 6(c)) showed the reduction of TXB_2_ after IPostC in healthy livers with the 8x20 sec intervention, in BDL livers only with the 4x60 sec intervention, and in TAA livers with both IPostC interventions. During the late phase of reperfusion (Figure 6(d)), TXB_2_ was decreased after warm ischemia using IPostC in healthy livers and in TAA livers with both interventions, whereas in BDL livers, it was decreased only with IPostC the 4x60 sec intervention.

## 4. Discussion

The aim of this study was to determine whether* ischemic postconditioning* (IPostC) could protect healthy, fibrotic, and cirrhotic livers from IRI after 90 min of warm ischemia. Therefore, liver fibrosis was induced using bile duct ligation, and liver cirrhosis was performed by application of thioacetamide (TAA). The BDL livers showed low fibrosis and severe bile duct proliferation accompanied by fibrotic septa throughout the parenchyma and moderate inflammation. These modifications are typical after bile duct ligation and are characteristics of liver fibrosis [22]. After the administration of TAA via drinking water for 18 weeks, moderate fibrosis with the proliferation of fibroblasts and focally confluent septa developed in the livers. Additionally, the livers showed moderate inflammation and bile duct proliferation, as previously described [20, 23]. Based on the histological evaluation, the protective effect of IPostC is difficult to estimate. A histological illustration of the livers is quite inhomogeneous; thus, the comparison of the control groups with the intervention groups shows no clear difference. In the present manuscript, the intervention of ischemic postconditioning has been investigated for the first time in fibrotic and cirrhotic livers. Liver cirrhosis or fibrosis was induced by thioacetamide over 18 weeks or bile duct ligation for 4 weeks. The histological investigations confirmed liver fibrosis and liver cirrhosis with fibrotic septa and single cell necrosis. Liver cirrhosis itself demonstrates a wide spectrum of histologic pictures. Due to the widely branched vascular network, the protective effect of IPostC probably does not reach all liver lobes equally. In HE staining, thus, we could not clearly distinguish between the control and the intervention groups. Inhomogeneity is also observed in patients with liver cirrhosis and also in earlier studies with the here involved methods to induce liver cirrhosis in rats [24, 25]. Also ischemia reperfusion injury illustrates a wide spectrum of histological pictures. The combination of preinjury by liver cirrhosis and damage by ischemia reperfusion multiplies the variety of histological appearance. Therefore to exactly score an improvement of the histological picture following ischemic postconditioning is not realistic. Another experimental study assessing IPostC with HE and immunohistological stainings showed reduced vacuolation, hepatocellular necrosis, and cellular infiltrations with the decreased expression of TNF*α* and intercellular adhesion molecule 1 (ICAM 1) in liver tissue after IPostC [26].

For implementation of warm ischemia, an isolated rat liver perfusion was performed with 90 min of ischemia at 37°C and 90 min of reperfusion. To evaluate the influence of IPostC on livers after warm ischemia, we investigated LDH as a parameter of cell damage, bile flow as a measure of organ function, and portal perfusion pressure to detect disturbances in microcirculation. The aim of the present study was to test the protective effect of ischemic postconditioning. LDH is an excellent marker of cell damage and for ischemia reperfusion injury [27, 28]. All experiments in this study were performed with isolated perfused livers. Therefore liver injury was investigated specifically. A decrease in LDH by ischemic postconditioning therefore means that injury related to the liver was attenuated. AST and ALT are mainly produced in hepatocytes. However, in this specific setting of isolated perfused livers, LDH might be even better because it also measures cell damage for nonparenchymal liver cells which are also very important for ischemia reperfusion injury [29, 30]. Due to the isolated liver perfusion system we can state definitively that the injury comes from the liver itself. Therefore only LDH measurements seem sufficient for the aim of the present study. In addition to LDH, we also decided to investigate thromboxane B_2_ as an indicator of inflammatory processes in association with reduced microcirculation as a result of ischemia and reperfusion.

After 90 min of warm ischemia, LDH efflux dramatically increased because of extensive cell death. Values for healthy livers were much higher than those for BDL and TAA livers. Perhaps, when compared with healthy livers, fibrotic and cirrhotic livers do not have enough intact cells to release LDH during reperfusion. The IPostC showed a protective effect after warm ischemia in isolated rat liver perfusions of healthy livers and in predamaged livers suggested by the reduced release of LDH and increased bile flow. This finding indicates that the easy and practical IPostC method is able to attenuate cell damage and improve organ function with reduced complications after resections of fibrotic or cirrhotic livers. With regard to LDH, the effect of IPostC is more pronounced in the late phase of reperfusion, which may be evidence of the long-term effects of IPostC. Therefore, IPostC might protect livers longer than the observed reperfusion time. To date, no data exist comparing the different cycles of IPostC in the liver. We found that different combinations of IPostC cycles have no impact on the results, because the effects of many short cycles (8x20 sec) and a few long cycles (4x60 sec) were approximately equal. However, the advantage of the 4x60 sec cycle includes reduced tissue injury around the clamped vessels. Therefore, a few long cycles are the preferred intervention scheme, and may also be preferred in clinical situations. The idea of comparing many short cycles with fewer long cycles was that up to now there was no consensus in the literature on how to perform the most effective intervention of ischemic postconditioning. Therefore it is a big advantage of this study to have data about the comparison of many short cycles and fewer long cycles. These data are novel and we found no difference in the present study for these two intervention shames. Fewer long cycles might be easier to perform and to preserve the vessels so that the clamp will be opened and closed again. To date, no data exist for fibrotic and cirrhotic livers regarding IPostC. Only two experimental studies assessing ischemic preconditioning (IPC) described a protective effect in cirrhotic mouse and rat livers suggested by reduced necrosis and apoptosis, decreased ALT and AST, and increased NO and superoxide dismutase (SOD) levels in the serum and liver tissue [31, 32].

During a follow-up study, we induced liver ischemia in two groups of rats (90 min warm ischemia with or without IPostC 4x60 sec) and collected their reperfusion perfusate, which we subsequently used to perfuse a second healthy rat liver. We found that the perfusate derived from the postconditioned rat group showed substantially reduced damage potential compared with the perfusate derived from the nonpostconditioned rat group. We speculate that IPostC reduces the release of liver damaging metabolites into the perfusate, thus critically debilitating IRI. This reduction of damaging metabolites may be caused by previously described cumulative triggers and the effects of the resulting metabolites and end effectors released after IPostC. Another effect of IPostC may be a dosed supply of oxygen: pH-dependent enzymes are activated step-by-step, and oxidative processes are suppressed or incomplete. This follow-up study was performed for the first time; therefore, there are no data with which to compare the results of this study.

One crucial result of liver damage during ischemia is disturbed microcirculation mediated by TXA_2_ during interactions with NO and endothelin [17, 18]. TXA_2_ is an arachidonic acid derivative with G-protein-mediated functions in different organs and cells [33]. In addition to the activation of platelets and vasoconstriction, which is responsible for the narrowing of liver sinusoids and reduced microcirculation after ischemia, TXA_2_ contributes to inflammatory processes by increasing the expression of adhesion molecules [33]. With regard to liver diseases, such as fibrosis and cirrhosis, TXA_2_ is involved in the pathogenesis of portal hypertension, which is a serious complication [19]. Therefore, we investigated the levels of TXB_2_, the inactive metabolite of TXA_2_, in the perfusate. After ischemia and reperfusion, more TXB_2_ was released. However, IPostC caused a reduction of the levels of TXB_2_ in the healthy, fibrotic, and cirrhotic livers. This reduction indicates a protective effect of IPostC after warm ischemia. This protective effect results from altered microcirculation in the liver because of the reduced formation of TXA_2_ accompanied by improved blood flow in hepatic sinusoids resulting in a better supply of oxygen and nutrients to hepatocytes. To date, no data exist regarding IPostC in fibrotic and cirrhotic livers; however, an experimental study assessing ischemic preconditioning (IPC) in liver cirrhosis described significantly increased values of NO in serum after IPC [32]. Nitric oxide functions as a vasodilator and improves blood flow in sinusoids, thus suggesting an effect of IPC on microcirculation. Besides TXA_2_ and NO autophagy might be an additional mechanism involved in the protective effects of ischemic postconditioning. Relevant novel studies support the role of autophagy in reducing ischemia reperfusion injury. Recently the protective effect of ulinastatin was described, at least in part due to autophagy activation [34]. The mechanisms found in the present study are related to a decreased production of damaging metabolites following protection by ischemic postconditioning. These data were generated by our so called follow-up studies where we used the medium of a first animal to perfuse a second liver. The damaging potential of the ischemic postconditioned medium was much lower. Autophagy activation might be possible but probably of minor relevance in this setting.

In summary, IPostC offers a very promising method to protect healthy, fibrotic, and cirrhotic livers from IRI. From a clinical point of view, this intervention may help to reduce complications and to improve graft function after liver resections.

## Figures and Tables

**Table 1:** Histological evaluation of healthy, fibrotic, and cirrhotic livers after warm ischemia ± IPostC.

Liver model	IRI-parameter	Comments
Healthy	Fibrosis: 0	
	Inflammation: 1	single cell necroses; apoptosis; mitoses
	Bile duct proliferation: 0	
	Fat deposition: 2	microvesicular
	Group necroses: 2	diffuse; cells without clear core loss, but with loss of color; loosened cytoplasm

BDL	Fibrosis: 1	septa in the area of bile duct proliferation
	Inflammation: 2	Single cell necroses; apoptosis; mitoses
	Bile duct proliferation: 3	diffusely distributed throughout the parenchyma
	Fat deposition: 1	very low microvesicular
	Group necroses: 1	

TAA	Fibrosis: 2	proliferation of fibroblasts; focally confluent septa
	Inflammation: 2	mixed; predominantly chronic focal suppurative-destructive cholangitis; single cell necroses; apoptosis; mitoses
	Bile duct proliferation: 2	plus accumulation of proliferations of vessels and fibroblasts
	Fat deposition: 1	very low microvesicular
	Group necroses: 1	focally with beginning connective tissue remodeling

## Data Availability

The data (Excel) used to support the findings of this study are available from the corresponding author upon request.

## Abbreviations

BDL: Bile duct ligation
EvG: Elastica-van-Gieson
HE: Haematoxylin/eosin
ICAM: Intercellular adhesion molecule
IPC: Ischemic preconditioning
IPostC: Ischemic postconditioning
IRI: Ischemia reperfusion injury
LDH: Lactate dehydrogenase
NO: Nitric oxide
SOD: Superoxide dismutase
TAA: Thioacetamide
TX: Thromboxane.

## Supplementary Materials

Supplementary Materials Normality test of portal perfusion pressure (Supplementary Table 1A) and LDH (Supplementary Table 1B) values after ischemia were calculated by Pearson's chi-squared test.

## References

[1] Mendes-Braz M., Elias-Miró M., Jiménez-Castro M. B., Casillas-Ramírez A., Ramalho F. S., Peralta C. (2012). The current state of knowledge of Hepatic Ischemia-ReperfusionInjury Based on Its Study in Experimental Models. *Journal of Biomedicine and Biotechnology*.

[2] Abu-Amara M., Gurusamy K. S., Hori S., Glantzounis G., Fuller B., Davidson B. R. (2009). Pharmacological interventions for ischaemia reperfusion injury in liver resection surgery performed under vascular control. *Cochrane Database of Systematic Reviews*.

[3] Saidi R. F., Chang J., Verb S. (2007). The effect of methylprednisolone on warm ischemia-reperfusion injury in the liver. *The American Journal of Surgery*.

[4] Soltys K., Dikdan G., Koneru B. (2001). Oxidative stress in fatty livers of obese Zucker rats: Rapid amelioration and improved tolerance to warm ischemia with tocopherol. *Hepatology*.

[5] Murry C. E., Jennings R. B., Reimer K. A. (1986). Preconditioning with ischemia: a delay of lethal cell injury in ischemic myocardium. *Circulation*.

[6] Gurusamy K. S., Kumar Y., Pamecha V., Sharma D., Davidson B. R. (2009). Ischaemic pre-conditioning for elective liver resections performed under vascular occlusion. *Cochrane Database of Systematic Reviews*.

[7] Baxter G. F., Yellon D. M. (2003). Current trends and controversies in ischemia-reperfusion research--meeting report of the hatter institute 3rd international workshop on cardioprotection. *Basic Research in Cardiology*.

[8] Tsang A., Hausenloy D. J., Mocanu M. M., Yellon D. M. (2004). Postconditioning: a form of ‘modified reperfusion’ protects the myocardium by activating the phosphatidylinositol 3-kinase-Akt pathway. *Circulation Research*.

[9] Staat P., Rioufol G., Piot C. (2005). Postconditioning the human heart. *Circulation*.

[10] Zhao C. M., Yang X. J., Yanga J. H. (2012). Effect of ischaemic postconditioning on recovery of left ventricular contractile function after acute myocardial infarction. *Journal of International Medical Research*.

[11] Freixa X., Bellera N., Ortiz-Pérez J. T. (2012). Ischaemic postconditioning revisited: lack of effects on infarct size following primary percutaneous coronary intervention. *European Heart Journal*.

[12] Hahn J. Y., Song Y. B., Kim E. K. (1889). Ischemic postconditioning during primary percutaneous coronary intervention: the effects of postconditioning on myocardial reperfusion in patients with ST-segment elevation myocardial infarction (POST) randomized trial. *Circulation*.

[13] Sun K., Liu Z.-S., Sun Q. (2004). Role of mitochondria in cell apoptosis during hepatic ischemia-reperfusion injury and protective effect of ischemic postconditioning. *World Journal of Gastroenterology*.

[14] Young S. B., Pires A. R. C., Boaventura G. T., Ferreira A. M. R., Martinho J. M. S. G., Galhardo M. A. (2014). Effect of ischemic preconditioning and postconditioning on liver regeneration in prepubertal rats. *Transplantation Proceedings*.

[15] Knudsen A. R., Kannerup A.-S., Dich R. (2012). Ischemic pre- and postconditioning has pronounced effects on gene expression profiles in the rat liver after ischemia/reperfusion. *American Journal of Physiology-Gastrointestinal and Liver Physiology*.

[16] Selzner N., Boehnert M., Selzner M. (2012). Preconditioning, postconditioning, and remote conditioning in solid organ transplantation: basic mechanisms and translational applications. *Transplantation Reviews*.

[17] Peralta C., Closa D., Hotter G., Gelpí E., Prats N., Roselló-Catafau J. (1996). Liver ischemic preconditioning is mediated by the inhibitory action of nitric oxide on endothelin. *Biochemical and Biophysical Research Communications*.

[18] Peralta C., Jiménez-Castro M. B., Gracia-Sancho J. (2013). Hepatic ischemia and reperfusion injury: effects on the liver sinusoidal milieu. *Journal of Hepatology*.

[19] Steib C. J., Gerbes A. L., Bystron M. (2007). Kupffer cell activation in normal and fibrotic livers increases portal pressure via thromboxane A2. *Journal of Hepatology*.

[20] Laleman W., Elst I. V., Zeegers M. (2006). A stable model of cirrhotic portal hypertension in the rat: Thioacetamide revisited. *European Journal of Clinical Investigation*.

[21] Arab J. P., Martin-Mateos R. M., Shah V. H. (2018). Gut–liver axis, cirrhosis and portal hypertension: the chicken and the egg. *Hepatology International*.

[22] Marques T. G., Chaib E., da Fonseca J. H. (2012). Review of experimental models for inducing hepatic cirrhosis by bile duct ligation and carbon tetrachloride injection. *Acta Cirurgica Brasileira*.

[23] Steib C. J., Bilzer M., Op Den Winkel M. (2010). Treatment with the leukotriene inhibitor montelukast for 10 days attenuates portal hypertension in rat liver cirrhosis. *Hepatology*.

[24] Yoon J. H., Lee J. M., Kim E., Okuaki T., Han J. K. (2017). Quantitative liver function analysis: Volumetric T1 mapping with fast multisection B1 inhomogeneity correction in hepatocyte-specifc contrastenhanced liver MR imaging. *Radiology*.

[25] Liedtke C., Luedde T., Sauerbruch T. (2013). Experimental liver fibrosis research: update on animal models, legal issues and translational aspects. *Fibrogenesis & Tissue Repair*.

[26] Guo J. Y., Yang T., Sun X. G. (2011). Ischemic postconditioning attenuates liver warm ischemia-reperfusion injury through Akt-eNOS-NO-HIF pathway. *Journal of Biomedical Science*.

[27] Hlaváčová M., Olejníčková V., Ronzhina M. (2017). Tolerance of isolated rabbit hearts to short ischemic periods is affected by increased LV mass fraction. *Physiological Research*.

[28] Karaca P., Konuralp C., Enç Y. (2006). Cardioprotective effect of aprotinin on myocardial ischemia/reperfusion injury during cardiopulmonary bypass. *Circulation Journal*.

[29] Alchera E., Imarisio C., Mandili G. (2015). Pharmacological preconditioning by adenosine A2a receptor stimulation: features of the protected liver cell phenotype. *BioMed Research International*.

[30] Kuboki S., Shin T., Huber N. (2008). Peroxisome proliferator-activated receptor-*γ* protects against hepatic ischemia/reperfusion injury in mice. *Hepatology*.

[31] Jang J. H., Kang K.-J., Kang Y., Lee I.-S., Graf R., Clavien P.-A. (2008). Ischemic preconditioning and intermittent clamping confer protection against ischemic injury in the cirrhotic mouse liver. *Liver Transplantation*.

[32] Yong J., Bo Y., Bao-qiang W., Jian-jun T., Zhen Q. (2013). The optimal time window of ischemic preconditioning (IPC) on the reperfusion injury in moderate to severe hepatocirrhosis in rats. *Annals of Clinical & Laboratory Science*.

[33] Nakahata N. (2008). Thromboxane A_2_: physiology/pathophysiology, cellular signal transduction and pharmacology. *Pharmacology & Therapeutics*.

[34] Zhao Y., Cai H., Zhou P., Lin S., Pan Y., Liang X. (2019). Protective effect of ulinastatin on hepatic ischemia reperfusion injury through autophagy activation in Chang liver cells. *Journal of Cellular Biochemistry*.

