# Supplementary material for: Ischemic Postconditioning (IPostC) Protects Fibrotic and Cirrhotic Rat Livers after Warm Ischemia
Source: Can J Gastroenterol Hepatol. 2019 Jun 9;2019:5683479. doi: 10.1155/2019/5683479 (PMC6590494; doi:10.1155/2019/5683479)
Supplement: Supplementary Materials — Normality test of portal perfusion pressure (Supplementary Table 1A) and LDH (Supplementary Table 1B) values after ischemia were calculated by Pearson's chi-squared test. [file 5683479.f1.docx]

Supplementary Table. 1A: Normality test of portal perfusion pressure values after ischemia

| Time after ischemia (min) | Healthy | | BDL 4 weeks | | TAA 18 weeks | |
| --- | --- | --- | --- | --- | --- | --- |
|  | **K2** | **P value** | **K2** | **P value** | **K2** | **P value** |
| 1 | 5.04 | 0.0805 | 0.7729 | 0.6795 | 3.119 | 0.2102 |
| 3 | 0.7853 | 0.6753 | 2.073 | 0.3547 | 1.847 | 0.3972 |
| 6 | 1.103 | 0.5761 | 2.106 | 0.3489 | 0.1131 | 0.945 |
| 9 | 1.33 | 0.5142 | 3.672 | 0.1594 | 0.08789 | 0.957 |
| 20 | 0.8157 | 0.6651 | 6.549 | 0.0378 | 0.4783 | 0.7873 |
| 30 | 1.701 | 0.4271 | 3.211 | 0.2008 | 0.09547 | 0.9534 |
| 40 | 1.648 | 0.4387 | 1.883 | 0.3901 | 0.8615 | 0.65 |
| 50 | 1.296 | 0.523 | 1.215 | 0.5448 | 0.4768 | 0.7879 |
| 60 | 0.3703 | 0.831 | 1.333 | 0.5134 | 0.1163 | 0.9435 |
| 70 | 2.926 | 0.2315 | 1.233 | 0.5397 | 0.1136 | 0.9448 |
| 80 | 0.3236 | 0.8506 | 1.794 | 0.4077 | 0.04779 | 0.9764 |
| 90 | 0.4407 | 0.8022 | 1.005 | 0.6051 | 0.07662 | 0.9624 |

Normality of portal perfusion pressure values’ distribution was tested by Pearson's chi-squared test, P value>0.05 was considered as normality distribution.

Supplementary Table. 1B: Normality test of LDH values after ischemia

| Time after ischemia (min) | Healthy | | BDL 4 weeks | | TAA 18 weeks | |
| --- | --- | --- | --- | --- | --- | --- |
|  | **K2** | **P value** | **K2** | **P value** | **K2** | **P value** |
| 1 | 2.431 | 0.2965 | 4.782 | 0.0916 | 0.2092 | 0.9007 |
| 3 | 1.526 | 0.4662 | 1.778 | 0.411 | 3.516 | 0.1724 |
| 6 | 2.237 | 0.3268 | 3.323 | 0.1898 | 9.631 | 0.0081 |
| 9 | 3.127 | 0.2094 | 2.829 | 0.2431 | 16.95 | 0.0002 |
| 20 | 3.37 | 0.1854 | 0.5089 | 0.7753 | 12.56 | 0.0019 |
| 30 | 2.905 | 0.234 | 2.882 | 0.2367 | 12.98 | 0.0015 |
| 40 | 1.334 | 0.5133 | 1.809 | 0.4048 | 9.81 | 0.0074 |
| 50 | 2.497 | 0.287 | 3.052 | 0.2175 | 1.443 | 0.4861 |
| 60 | 1.802 | 0.4061 | 1.926 | 0.3818 | 1.412 | 0.4937 |
| 70 | 0.7472 | 0.6882 | 2.922 | 0.232 | 0.3032 | 0.8593 |
| 80 | 1.806 | 0.4054 | 2.695 | 0.2599 | 0.1044 | 0.9491 |
| 90 | 0.1351 | 0.9347 | 2.201 | 0.3326 | 0.4218 | 0.8098 |

Normality of LDH values’ distribution was tested by Pearson's chi-squared test, P value>0.05 was considered as normality distribution.
